# Photolithographic patterning of cellulose: a versatile dual-tone photoresist for advanced applications

**DOI:** 10.1007/s10570-014-0471-4

**Published:** 2014-10-16

**Authors:** Archim Wolfberger, Andreas Petritz, Alexander Fian, Jakob Herka, Volker Schmidt, Barbara Stadlober, Rupert Kargl, Stefan Spirk, Thomas Griesser

**Affiliations:** 1 Chair of Chemistry of Polymeric Materials, University of Leoben, Otto Glöckel-Straße 2, 8700 Leoben, Austria; 2 Materials-Institute for Surface Technologies and Photonics, Joanneum Research Forschungsgesellschaft mbH, Franz-Pichler-Straße 30, 8160 Weiz, Austria; 3 Faculty of Mechanical Engineering, University of Maribor, Smetanova 17, 2000 Maribor, Slovenia; 4 Institute for Chemistry and Technology of Materials, Graz University of Technology, Stremayrgasse 9, 8010 Graz, Austria

**Keywords:** Cellulose, Photochemistry, Photoresist, Lithography, Organic electronics, Organic thin film transistor

## Abstract

In many areas of science and technology, patterned films and surfaces play a key role in engineering and development of advanced materials. Here, we present a versatile toolbox that provides an easy patterning method for cellulose thin films by means of photolithography and enzymatic digestion. A patterned UV-illumination of trimethylsilyl cellulose thin films containing small amounts of a photo acid generator leads to a desilylation reaction and thus to the formation of cellulose in the irradiated areas. Depending on the conditions of development, either negative and positive type cellulose structures can be obtained, offering lateral resolutions down to the single-digit micro meter range by means of contact photolithography. In order to highlight the potential of this material for advanced patterning techniques, cellulose structures with sub-µm resolution are fabricated by means of two-photon absorption lithography. Moreover, these photochemically structured cellulose thin films are successfully implemented as dielectric layers in prototype organic thin film transistors. Such photopatternable dielectric layers are crucial for the realization of electrical interconnects for demanding organic device architectures.

**Electronic supplementary material:**

The online version of this article (doi:10.1007/s10570-014-0471-4) contains supplementary material, which is available to authorized users.

## Introduction

Cellulose, as the most abundant biopolymer on earth and major component of green plants features a set of properties which can hardly be found in any other single material (Klemm et al. 2005). It has been used for ages by mankind to maintain information and knowledge (paper), to provide clothing (fibers) and to protect mankind against nature’s forces (wood) (Klemm et al. 2004). In the past two decades, also other cellulose based materials entered into the focus of interest, since progress in preparation and analysis of materials allowed scientists to move from the macro- and microscale to the nanoscale world. A wide variety of cellulose based materials such as nanofibrils, nanocrystals, nanofibers, nanoparticles, aerogels and ultrathin cellulose films have recently been explored, to give just a few examples (Schaub et al. 1993; Kontturi et al. 2003a, b; Eichhorn et al. 2010; Habibi et al. 2010; Moon et al. 2011; Olsson et al. 2010). The properties of these materials can be classified as unusual compared to those of macroscale cellulose materials. Particularly, the isolation of nanocrystalline moieties from bulk cellulose lead to a variety of non-classic applications of cellulose in charge storage for supercapacitors (Thielemans et al. 2009; Liew et al. 2010, 2013), high mechanical strength materials (Cranston et al. 2011), and optics (Cranston and Gray 2006, 2010). Lately, also large efforts have been made to use (nano)paper as a substrate for field effect transistors (Fortunato et al. 2008; Huang et al. 2013; Fujisaki et al. 2014), since paper provides several interesting properties such as low price, ready availability and excellent printability with organic polymers. Most notably, the combination of its mechanical properties, environmental stability and raw material availability makes cellulose an ideal candidate for environmentally sustainable and biocompatible products for a wide range of applications. One of the main disadvantages of cellulose, however, is its poor solubility in common organic solvents and therefore its constrained processability, limiting its applications especially in the growing field of organic electronics. In order to overcome these limitations, various procedures for the regeneration of cellulose from organosoluble cellulose derivatives have been developed, paving the way towards novel biodegradable functional materials (Klemm et al. 2005). A promising cellulose derivative for the preparation of cellulose thin films is trimethylsilyl cellulose (TMSC), which is soluble in several common organic solvents, including eco-friendly solvents such as ethanol and can be regenerated to cellulose by a treatment with vapors or solutions of hydrochloric acid (Rolland 1993; Kontturi et al. 2003b; Kontturi and Lankinen 2010). While such thin films have been widely employed to study and to understand the interaction of a variety of biomolecules with cellulose, micro- and macropatterned cellulose films have been shown to be promising materials for the fabrication of protein microarrays, high protein affinity matrices or for sensitive DNA detection (Löscher et al. 1998; Orelma et al. 2011, 2012; Mohan et al. 2013b, c). Blends on the basis of TMSC and other polymers such as styrene or lignins can lead to the formation of micro- and nanostructures as well due to phase separation (Nyfors et al. 2009; Hoeger et al. 2012). Although such structures may be used for sensor purposes (e.g. by selective immobilization of Au-nanoparticles), the major drawback of this method is that spatially resolved structures can hardly be realized (Taajamaa et al. 2013). Recently, TMSC has been successfully applied as a precursor for the fabrication of cellulose based high-k dielectric layers in pentacene- and fullerene (C_60_) based OTFTs, as demonstrated by our group (Petritz et al. 2013). However, for the realization of complex organic circuits, efficient patterning procedures for dielectric materials are of particular importance, in order to enable the fabrication of electrical interconnections. In many areas of research ranging from biosensors to lab-on-a-chip devices or organic electronics, there is a need for high-throughput production methods for microstructured cellulose surfaces. So far, methods to create such cellulose micropatterns are rather rare and include soft lithography and deep UV lithography, using UV-light with wavelengths below 260 nm, both of which have some disadvantages and limitations (Kargl et al. 2013; Tanaka et al. 2004). While the use of soft lithography is too laborious, the high energy input of deep UV lithography limits its usage in a variety of material fabrication processes (e.g. for organic thin film transistors). In addition, the cellulose patterns are created by photodegradation in the illuminated areas, which only allows for the realization of negative tone photoresists. A widely used concept for the fabrication of polymer micro structures is based on a photoinduced alteration of the solubility of polymeric materials. This concept is also applied in chemically amplified photoresists (CARs), which utilize photo acid generators (PAGs) to adjust the solubility by means of UV-light (Ito et al. 1990).

In this contribution, we present the photo-induced conversion of acid labile TMSC to rather insoluble cellulose with the aid of PAGs. Although CARs which exploit desilylation reactions are well known (Cunningham 1987; Cunningham et al. 1987), these methods have, to the best of our knowledge, not yet been used for the fabrication of patterned cellulose thin films from easily accessible TMSC. Moreover, the herein described approach enables the realization of both positive and negative type microstructured cellulose thin films, following the concept of dual-tone photoresists. Going a step beyond conventional lithographic techniques, two-photon absorption (TPA) lithography has been successfully applied to realize feature sizes in the sub-µm range. To demonstrate the versatility of this biopolymer based photoresist towards potential applications in organic electronics, this material has also been investigated as a photo-patternable ultrathin dielectric layer for low-voltage pentacene based OTFTs.

## Experimental

### Materials

Unless otherwise stated, all chemicals were obtained from commercial sources and were used without further purification. Trimethylsilyl cellulose with a degree of substitution of DS_Si_ = 2.8 was provided by the Thuringian Institute of Textile and Plastics Research (Rudolstadt, Germany). *N-*hydroxynaphthalimide triflate (electronic grade, ≥99 %) and cellulase from *Trichoderma viride* were obtained from Sigma Aldrich. Silicon wafers were obtained from Taisil Electronic Materials Corp. and were rinsed with acetone and cleaned with a polymer cleaning solution (First Contact, Photonic Cleaning Technology, LLC) after cutting.

### Sample preparation

TMSC films were fabricated by spin coating from chloroform solutions with concentrations ranging from 5 to 20 mg ml^−1^ (v = 2,000 rpm, a = 1,000 rpm s^−1^) containing varying amounts of photoacid generator onto silicon wafers or CaF_2_ plates.

### UV-irradiation

UV-irradiation experiments were carried out with a medium pressure Hg-lamp (100 W, Newport, 66990) equipped with a filter transmissive for wavelengths in the range of 350–450 nm. The light intensity (power density) at the sample surface was measured with a UV radiometer (UV Power Puck, EIT, Inc.) and was determined as 7.6 mW cm^−2^ in the spectral range from 250 to 390 nm (UV-A, UV-B and UV-C). Photolithographic patterning was carried out with a mask aligner (500 W HgXe, SUSS, MJB4) equipped with a filter transmissive for wavelengths in the range of 365 nm with a measured power density of 9.0 mW cm^−2^.

### TPA lithography

For all TPA lithography experiments, a commercial lithography setup (Photonic Professional, Nanoscribe GmbH) was used. A laser power of 15 mW and a lateral feed rate of 50 µm s^−1^ with a 100× oil immersion objective with NA = 1.4 and a tight focusing of the laser beam were chosen.

### Development

After photolithographic patterning, a development was performed in chloroform for 10 min at room temperature or via enzymatic digestion using cellulase from *T. viride* (1 mg ml^−1^, dissolved in a 100 mM sodium acetate/acetic acid buffer at pH 4.8). The illuminated samples were immersed in 3–5 ml of cellulase solution at 37 °C overnight.

### FTIR spectroscopy

FTIR spectra were recorded on a Perkin Elmer Spectrum One instrument (spectral range of 850–4,000 cm^−1^, resolution of 1 cm^−1^) in transmission mode on CaF_2_ plates.

### Atomic force microscopy

Atomic force microscopy (AFM) micrographs were recorded with a Nanosurf FlexAFM instrument, using silicon AFM probes with a resonance frequency of 190 kHz and a force constant of 48 N m^−1^ (Tap190AL-G, Budgetsensors).

### Device fabrication

Organic thin film transistors were fabricated in a staggered bottom-gate top-contact architecture. The gate electrode was processed on pre-cleaned glass slides by thermal evaporation of a 40 nm thick aluminum layer through a shadow mask at a rate of 1 nm s^−1^ under high vacuum conditions. For the negative type photolithographic patterning of TMSC, a metal shadow mask was used. After spin-coating and patterning (UV-irradiation and development) of the dielectric, a 35 nm thick pentacene layer was evaporated. Source- and drain electrodes were deposited by thermal evaporation of gold through a shadow mask in order to form 50 nm thick contacts. After production, all OTFT samples were protected from light and stored under argon atmosphere. OTFTs were fabricated with a channel-length of 70 µm and width of 1.5 mm.

### Electrical characterization

The dielectric properties of the dielectric were determined by frequency dependent capacitance (C-f) and current–voltage (I-V) measurements on metal (30 nm Al)—cellulose films (photochemical regenerated TMSC films, 32 nm)—metal (50 nm Al) sandwich structures with an overlap area of 0.1 cm^2^ on glass substrates. The frequency dependence of the gate dielectric capacitance was measured by impedance spectroscopy techniques with an LCR meter (Hioki 3532-50 LCR). For the data processing of the OTFT characteristics, the capacitance at 1 kHz was used. Electrical measurements of the OTFTs were carried out under exclusion of light, using a parameter analyzer from MB-Technologies.

## Results and discussion

### Investigation of the photoreaction

**Scheme 1:**
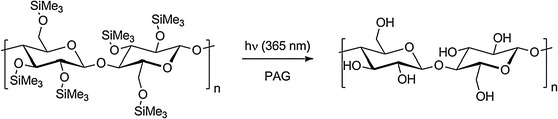
Photo-acid induced desilylation reaction of TMSC

For the investigation of the photoinduced desilylation reaction, thin films of TMSC containing 2 wt% of the non-ionic photoacid generator *N*-hydroxynaphthalimide triflate (NHNA) were prepared by spin coating onto CaF_2_ plates, leading to films with a thickness of approximately 190 nm. In order to prevent a photodegradation of the material, which preferentially occurs at wavelengths of λ ≤254 nm, UV-irradiation was carried out under nitrogen atmosphere at wavelengths higher than 300 nm. UV-exposure of a TMSC/NHNA blend yields triflic acid as the main photoproduct, which subsequently causes a cleavage of the trimethylsilyl (TMS) groups, resulting in a conversion of TMSC to cellulose as depicted in Scheme 1.

**Fig. 1:**
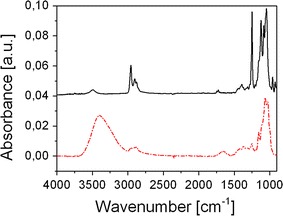
FTIR spectra of TMSC, containing 2 wt% NHNA on CaF_2_ plates before illumination (*black solid line*) and after illumination (*red dotted line* E = 5.2 J cm^−2^). (Color figure online)

The photoinduced desilylation was followed by means of FTIR spectroscopy. In Fig. 1, the obtained FTIR spectra of thin TMSC/NHNA films prior to and after exposure to UV-light (E = 5.2 J cm^−2^) are displayed. The FTIR spectrum before UV-illumination shows a weak signal at 3,490 cm^−1^, which can be assigned to residual hydroxyl moieties stemming from an incomplete silylation of the hydroxyl moieties as expected for TMSC with a degree of substitution (DS_Si_) of 2.8, which was used for this study. UV-illumination leads to a significant increase of the O–H stretching vibration, while the intensities of the Si–C rocking vibrations at 1,250 cm^−1^ and C–H stretching vibrations at 2,957 cm^−1^ decreased, which is consistent with the proposed desilylation reaction in Scheme 1. The observed changes in the FTIR spectra reveal a nearly complete conversion of TMSC to cellulose. These findings were also supported by X-ray photoelectron spectroscopy (XPS). In accordance with the FTIR study, C_1s_ detail spectra (shown in the supporting information) exhibit a significantly lower Si–C signal at 284.6 eV in relation to the C–O signal at 286.9 eV after illumination. Additionally a decrease in the total Si content from 11.7 to 2.7 at.% at the sample surface can be observed, which additionally confirms the UV-induced desilylation reaction. The photo-generated hydroxyl groups influence the surface energy of the TMSC/NHNA layers towards a higher polarity, which has already been observed for desilylation of TMSC using vapors of hydrochloric acid (Mohan et al. 2011, 2012). A study describing this behavior in detail can be found in the supporting information.

**Fig. 2:**
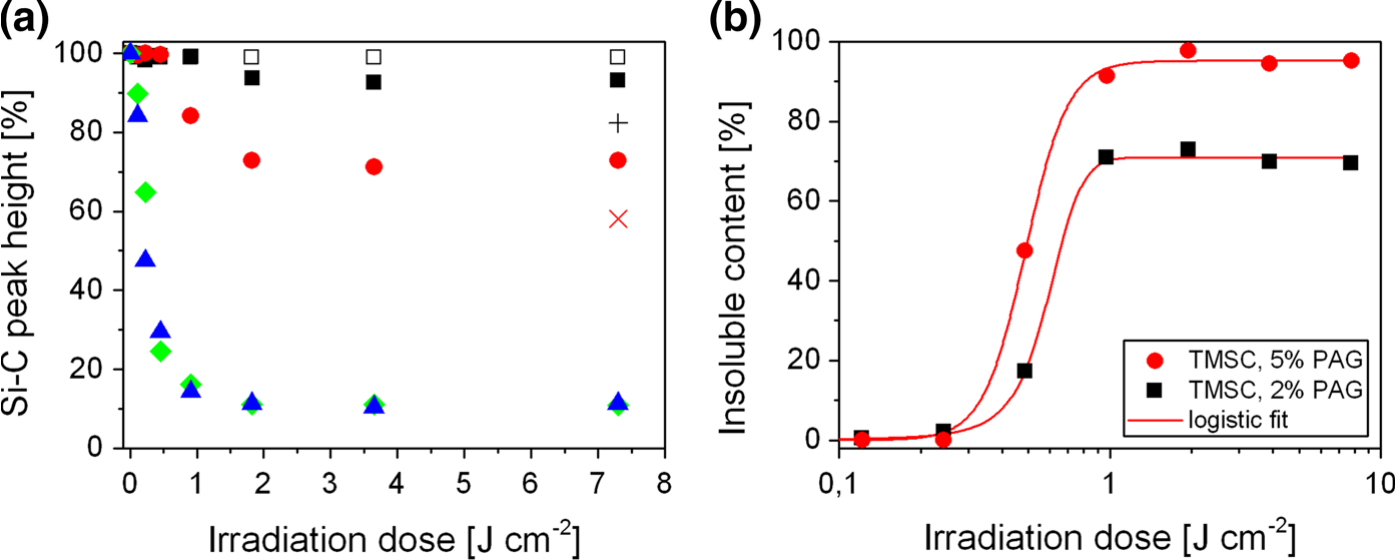
**a** Photoinduced depletion of the silyl ether groups in TMSC films, containing no PAG (*open squares*), 1 wt% NHNA (*black squares*), 2 wt% NHNA (*red circles*), 5 wt% NHNA (*green diamonds*) and 10 wt% NHNA (*blue triangles*) during UV-illumination and 24 h after illumination for 1 wt% NHNA (*black plus sign*) and 2 wt% NHNA (*red cross sign*); **b** gel-fraction (insoluble fraction) of TMSC films containing 2 wt% NHNA (*black squares*) and 5 wt% NHNA (*red circles*) during UV-illumination and after a development in chloroform. (Color figure online)

Furthermore, it was found that the amount of NHNA present in the TMSC films significantly influences the conversion of TMSC to cellulose. The kinetic behavior of the desilylation reaction for different PAG concentrations was determined by evaluating the decrease of the Si–C band during UV-illumination as shown in Fig. 2a. While a UV exposure of TMSC, containing 5 and 10 wt% NHNA leads to an almost complete desilylation (approx. 11 % remaining silyl ether groups after illumination with E ≥1.8 J cm^−2^), lower NHNA concentrations of 1 and 2 wt%, lead to an insufficient conversion of 7 and 27 %, respectively, after a prolonged illumination with an irradiation dose of E = 7.3 J cm^-2^. TMSC films without photoacid generator show no decrease of the Si–C band, leading to the conclusion that an UV induced cleavage of the silyl ether bond does not occur without an additional PAG component.

It has to be mentioned that the desilylation reaction also proceeds in the TMSC films after UV-irradiation. This phenomenon is already known from cationic photo-polymerization reactions and is referred to as “dark reaction”. In this case, the photo-generated protons catalyze the desilylation reaction in absence of UV-light. This reaction causes a further decrease of the silyl ether content from 93 to 82 % and from 73 to 58 % in TMSC films containing 1 and 2 wt% NHNA, respectively, after storage under exclusion of light for 24 h as depicted in Fig. 2a. FTIR spectra, recorded after 48 h, did not exhibit any further decrease of the Si–C band.

### Photolithographic patterning of TMSC

#### Negative type development

Due to the fact that the photo-generated cellulose is insoluble in common organic solvents such as chloroform or toluene, a direct photolithographic patterning of TMSC, containing small amounts of PAG is possible, yielding negative type cellulose structures after development. The changes in solubility of TMSC films, caused by the photoinduced desilylation reaction were assessed by means of sol–gel analysis. The insoluble fraction (gel fraction) was determined by FTIR spectroscopy by evaluating the intensity of the C–O–C stretching vibration of the glycosidic bond at 1,150–1,170 cm^−1^ and comparing the peak height before and after development in chloroform for 10 min. Figure 2b represents the gel fraction of TMSC-films containing 2 wt% NHNA and 5 wt% NHNA as a function of the irradiation dose, revealing maximum gel fractions of 73 and 98 %, respectively, after UV-illumination with an irradiation dose of E ≥0.97 J cm^−2^. Prolonged exposure does not further influence the gel fraction, which is in good accordance with the kinetic behavior shown in Fig. 2a. A decrease in solubility after an additional exposure (irradiation doses up to 70 J cm^−2^) could not be observed, which excludes a photodegradation of the cellulose backbone. Compared to commercially available photoresists, the required illumination doses in the range of 1 J cm^−2^ are rather high for photolithographic patterning. This can be attributed to an insufficient spectral overlap of the UV absorption spectrum of NHNA and the used polychromatic irradiation source (as displayed in the supporting information). It can be assumed that a better matching of the spectral overlap leads to a reasonable photoresist performance. With respect to a possible application of these films as a dielectric material in organic thin film transistors, further photo-patterning experiments were performed with TMSC films, containing 2 wt% NHNA. Although, this concentration leads to an incomplete conversion of the TMSC to cellulose, the changes in solubility are sufficient for a successful photopatterning. Higher PAG contents (and their ionic photocleavage products) may also negatively influence the device stability, therefore we aimed for a compromise between low PAG concentration and a high obtainable gel fraction. A NHNA concentration of 2 wt% was found to be ideal in this respect.

#### Positive type development

**Fig. 3:**
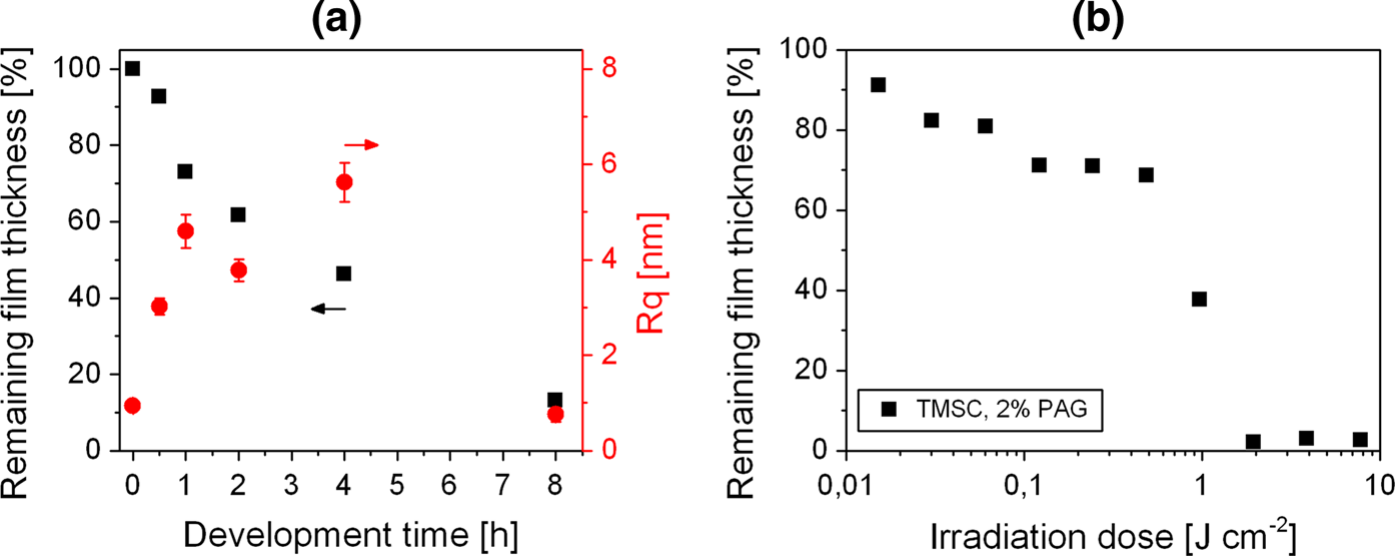
**a** Remaining film thickness and rms roughness of UV-illuminated TMSC films (E = 4.6 J cm^−2^) after different durations of enzymatic digestion; **b** remaining film thickness of UV-illuminated TMSC films, containing 2 wt% NHNA after development in enzyme solution

Going a step beyond conventional resist development using organic solvents, enzymatic digestion has been evaluated for the realization of positive type cellulose structures. It is well known that cellulose can be digested by appropriate enzymes, e.g. cellulase from *T. viride* (Kargl et al. 2013; Mohan et al. 2013a; Ahola et al. 2008). Utilizing this fact, the selective enzymatic digestion of photo-regenerated cellulose has been investigated. Consequently, the illuminated films were immersed in an acetate buffer solution containing a mixture of cellulose digesting enzymes. For a complete enzymatic development, the kinetic behavior of digestion has been determined by measuring the decrease in the film thickness of UV-illuminated TMSC films (E = 4.6 J cm^−2^), prepared on silicon wafers, for different periods of time of enzymatic digestion. The determination of the remaining film thickness by means of FTIR spectroscopy was not applicable in this case, because the glycosidic bonds which were used for the previous sol–gel analysis are expected to be hydrolyzed due to enzyme activity. Since other FTIR signals were not found to be suitable for a sol–gel analysis, AFM was employed to characterize the films, exhibiting a film thickness of approximately 180 nm before UV-illumination. Figure 3a shows the remaining film thickness (related to the film thickness after illumination) as a function of the immersion time. It turned out that an almost complete digestion (4 % of the original film thickness) is achieved after 8 h of immersion. Moreover, the rms roughness R_q_ of the TMSC films significantly increases during enzymatic digestion and decreases again after 8 h of immersion in enzyme solution, indicating that only some residue of original film is remaining on the surface. In a control experiment, a degradation of non-illuminated TMSC films, containing 2 wt% NHNA could not be observed, demonstrating the selectivity of the used enzymes. In order to determine the sensitivity of this positive type photoresist in combination with an enzymatic development, a sol–gel analysis has been performed. Consequently, TMSC films, containing 2 wt% NHNA were illuminated for different periods of time and developed by an enzymatic treatment (16 h of immersion at room temperature). In Fig. 3b, the remaining film thickness (determined by means of AFM) of the illuminated TMSC film is plotted as a function of the illumination dose. The graph shows a pronounced threshold at an irradiation dose of 0.49 J cm^−2^ (69 % remaining thickness), leading to a complete digestion after an illumination dose of 1.9 J cm^−2^. These findings reveal that also partially regenerated cellulose (Si–C band intensity reveals a DS_Si_ of approximately 0.5 after irradiation) is digested by the used enzymes, while pristine TMSC (DS_Si_ = 2.8) is not affected at all. Although it appears surprising on first glance, the digestion of lowly substituted celluloses (e.g. cellulose acetate) has been already described in literature some time ago in the context of biodegradability of cellulosic materials (Reese 1957). In order to determine if the photogenerated acid in the illuminated films can also lead to a formation of water-soluble degradation products, TMSC films, containing 2 wt% NHNA were illuminated (E = 4.6 J cm^−2^) and immersed in buffer solution for an extended period of time without adding any enzyme. It was observed that the regenerated cellulose in the illuminated areas remained on the substrates, even after 72 h of immersion in acetate buffer.

#### Contact photolithography

**Fig. 4:**
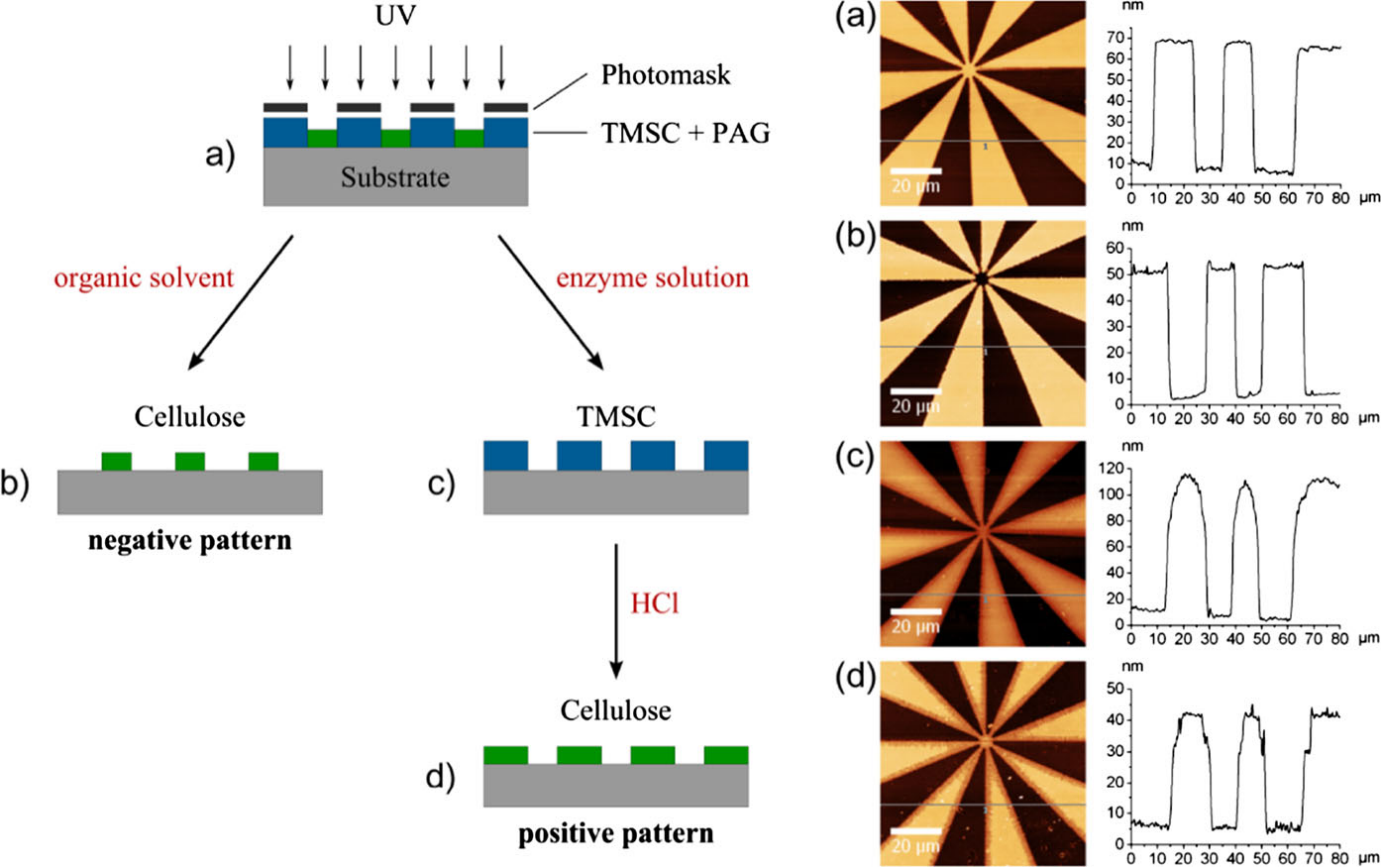
Overview on the photolithographic patterning process to obtain positive and negative type cellulose patterns (*blue* TMSC + NHNA, *green* cellulose) and corresponding AFM topography images and line profiles after **a** photolithographic patterning; **b** subsequent negative type development with chloroform; **c** positive development using enzymes and **d** after conversion to cellulose. (Color figure online)

In order to demonstrate the applicability of this material as a dual tone photoresist, thin TMSC films, containing 2 wt% NHNA were photopatterned on a mask aligner system. After patterned UV-irradiation (E = 5.4 J cm^−2^), the development of the TMSC films was either performed in chloroform or, alternatively, in a cellulase solution as depicted in Fig. 4a–c. For the fabrication of positive type cellulose structures, patterned TMSC films were subsequently treated with vapors of hydrochloric acid for 90 s (Fig. 4d), leading to a complete regeneration (Petritz et al. 2013). In addition, the intensity of the signal of the glycosidic bond at 1,150–1,170 cm^−1^ did not change after HCl treatment, leading to the conclusion that no significant degradation occurred. The observed decrease in the film thickness after the acid induced conversion of TMSC to cellulose can be related to a change in the density of the film due to the formation of hydrogen bonds of the resulting cellulose. Although both approaches yield well-defined cellulose structures with lateral resolutions in the range of 1–2 µm, the enzymatic development results in less well-defined edges as shown in the cross section in Fig. 4c. This can be explained by the comparably poor resist behavior, which is also reflected by the sol–gel analysis in Fig. 3b. Additionally, the extended development time of 24 h as well as the subsequent treatment with hydrochloric acid can negatively influence the quality of the obtained structures. Accordingly, the surface roughness R_q_ of these films increases during the patterning procedure from 0.70 ± 0.04 nm (non-illuminated sample) to 1.82 ± 0.02 nm (after patterned illumination, enzymatic development and subsequent hydrochloric acid treatment). Detailed AFM micrographs, revealing the surface morphology prior to and after the patterning procedure can be found in the supporting information. A negative type development of the patterned films in chloroform does not influence the surface roughness significantly (R_q_ = 0.75 ± 0.05 nm after development).

#### Two-photon absorption (TPA) lithography

**Fig. 5:**
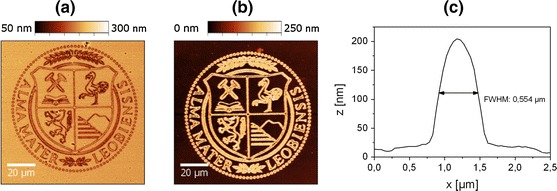
AFM micrographs of TMSC films, containing 10 wt% NHNA **a** after two-photon absorption lithography; **b** followed by a subsequent development in toluene and **c** corresponding line profile of a *single line*

Advancing from micro- to submicrometer resolutions, TPA lithography was evaluated for the fabrication of cellulose patterns. The two-photon excitation technique has attracted interest due to its intrinsic 3D processing capability and is considered as a promising technology with unique advantages regarding nanofabrication, enabling lateral resolutions of less than 100 nm (Lee et al. 2008; Juodkazis et al. 2005). Because of the aforementioned negative effects of enzymatic digestion on the achievable resolution, a negative type development using organic solvents was chosen in these experiments. For sub-µm patterning, TMSC films containing 10 wt% NHNA were patterned on a commercial lithographic setup with a laser power of 15 mW and lateral feed rate of 50 µm s^−1^. In this patterning setup, the laser beam (λ = 780 nm, repetition rate 100 MHz, pulse width 150 fs) causes multi-photon absorption in its focus, leading to an energy transfer from the laser to the PAG which initiates the desilylation reaction. Figure 5a shows the obtained pattern directly after TPA lithography visualized by means of AFM. A subsequent development of the film in toluene for 15 min yields freestanding cellulose structures with a height of approx. 180 nm and a lateral resolution of approx. 550 nm (full width at half maximum, FWHM) as shown in Fig. 5b, c. One important prerequisite for a two-photon induced patterning process is a sufficient TPA efficiency, i.e. a high TPA cross section of the used photoinitator, which mainly depends on the delocalized *p*-electron system of the photoinitiator (Pucher et al. 2009). Although the used photoacid generator offers an appropriate UV absorption behavior that corresponds with the applied laser wavelength under two-photon conditions, the TPA activity of this commercially available PAG has not been determined in detail. It can be assumed that tailored photoinitiators which provide better TPA coefficients enable even higher resolutions, comparable to those reported for cationic photoresists (e.g. SU-8).

### Application of photopatterned cellulose films as OTFT gate dielectrics

**Fig. 6:**
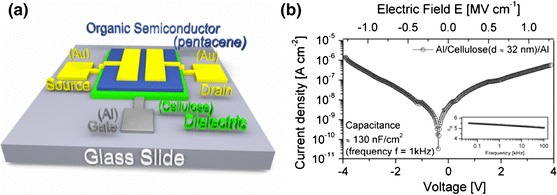
**a** Schematic image of fabricated OTFTs, showing the glass substrate (*light grey*), an aluminum gate electrode (*dark grey*), a cellulose film fabricated by negative photochemical regeneration of a TMSC film (*green*), a pentacene film as active layer (*dark blue*) and gold source-drain electrodes (*yellow*); **b** current density through a 32 nm thin cellulose film (negative photochemical regenerated TMSC film) as gate dielectrics in a capacitor structure with an overlap area of 0.1 cm^2^; *inset* in **b** shows frequency dependence of the dielectric constant ε_R_ of a thin photolithographically patterned cellulose film. (Color figure online)

In order to highlight a potential application for this cellulose based photoresist in the emerging area of organic electronics, photopatterned films were implemented as dielectric layers in low-voltage OTFTs with a device setup as shown in Fig. 6a. In general, patternable dielectric materials have to offer reasonable dielectric properties in addition to an appropriate resist behavior (in terms of sensitivity and resolution). For that purpose, the dielectric properties of 32 nm thin cellulose films, obtained by negative type photolithographic patterning of spin coated TMSC films, containing 2 wt% NHNA were investigated in capacitor structures. A current/voltage (I/V) measurement is plotted in Fig. 6b, revealing leakage currents in the order of 10^−6^ A cm^−2^ at an electric field of 1.3 MV cm^−1^, which is quite low, considering that a 32 nm thin patterned polymeric dielectric is used. A capacitance of approx. 130 nF cm^−2^ was measured, corresponding to a dielectric constant of ε_R_ = 5.4 ± 0.7 at 1 kHz with a sufficiently high frequency stability of ε_R_ up to 100 kHz (illustrated in the inset in Fig. 6b). The observed permittivity is significantly lower in comparison to the permittivity which we observed for cellulose films, fabricated by vapor phase acid hydrolysis in our previous work (ε_R_ = 8.4 ± 0.5) (Petritz et al. 2013). As already mentioned above, XPS as well as FTIR measurements reveal a remaining Si–C signal after the photochemical regeneration of cellulose which clearly indicates that the TMSC is not completely regenerated. In contrast, the cellulose films fabricated by vapor phase acid hydrolysis previously showed no remaining Si–C-signals and proved to be completely regenerated (Petritz et al. 2013). Therefore, a permittivity of 5.4 of the photochemically regenerated cellulose films, lying between fully regenerated cellulose (ε_R_ = 8.4) and pristine TMSC with a DS_Si_ of 2.8 (ε_R_ = 2.3) can be explained by the residuals of TMSC in the films.

**Fig. 7:**
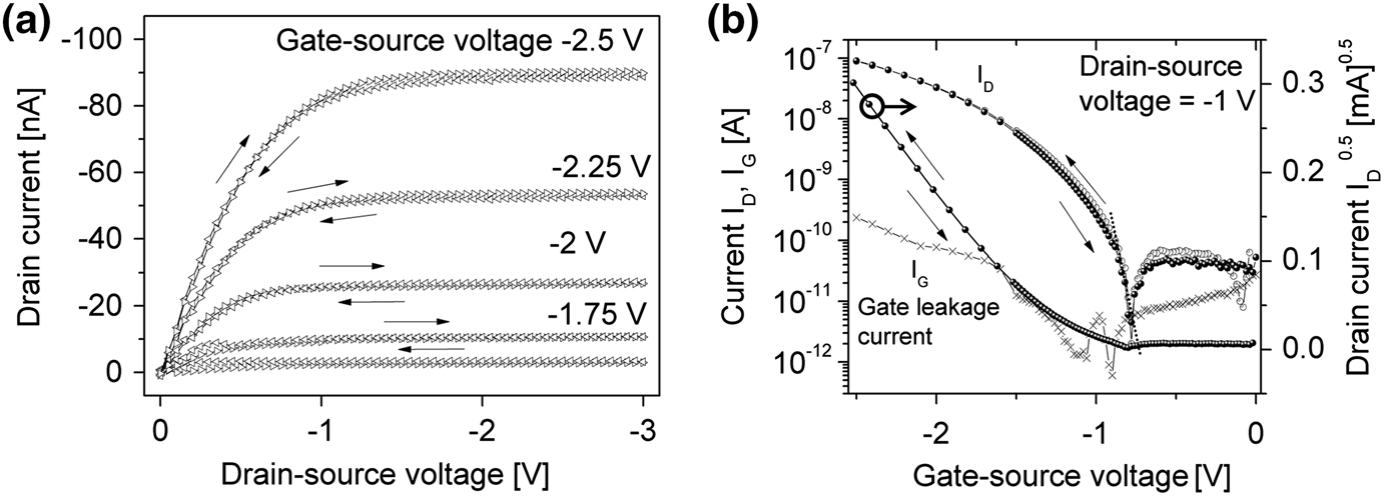
Electrical characteristics of pentacene based organic thin film transistor with a 32 nm thin cellulose film (negative type photolithograpically patterned TMSC film) as gate dielectric, featuring a channel length of 70 µm and channel width of 1.5 mm; **a** output characteristic and **b** transfer characteristics of the corresponding OTFT. The gate leakage current characteristics *I*_*G*_(*V*_*G*_) is also displayed

The electrical characteristics of a pentacene based OTFT with a negative type photolithographically patterned cellulose film as gate dielectric is plotted in Fig. 7. The output characteristics in Fig. 7a display a clear saturation of the drain current level with the gate bias. Furthermore, no hysteresis between forward and reverse drain voltage sweep is observed. In Fig. 7b the transfer characteristic is plotted, showing I_D_(V_G_) and I_G_(V_G_) in a semi-logarithmic representation as well as the square root of the drain current as a function of the gate bias. From the transfer characteristics the important performance parameters of the OTFT can be extracted, revealing an onset voltage V_on_ = −0.8 V, a threshold voltage of V_thr_ = −1.25 V and a subthreshold swing S as low as 110 mV dec.^−1^. The subthreshold swing S is the inverse of the maximum slope of the (quasi)linear part of the subthreshold current (dashed line in the semi-logarithmic plot of the transfer curve, as shown in Fig. 7b. Furthermore, the fabricated low voltage OTFTs show no hysteresis, gate leakage currents in the range of 80 pA, OFF-currents around 60 pA and a linear field effect mobility µ_lin_ of 0.08 cm^2^ Vs^−1^.

As a final remark we want to comment on the interface properties of our photopatternable dielectric material with the organic semiconductor in the OTFT, which is directly affecting the device performance. A low interface charge trap density is particularly essential for the fabrication of fast and stable organic electronic circuits. An upper limit of the density of interfacial trap states N_ss,max_ can be calculated from the obtained subthreshold swing according to the method reported by Rolland (1993). For the determined subthreshold swing of 110 mV dec.^−1^ an upper trap density limit of N_ss,max_ = 6.9 × 10^11^ cm^−2^ eV^−1^ is calculated. The extracted N_SS_ values for cellulose based OTFTs are exceptionally low and are in fact much smaller than the average interface trap density of states observed in amorphous silicon TFTs being in the range of 10^12^ cm^−2^ eV^−1^.

## Conclusions

In this contribution, we demonstrate a versatile method for an efficient photopatterning of cellulose thin films, the most abundant biopolymer on earth. Following the concept of dual-tone photoresist, it is possible to obtain either positive or negative type micropatterns depending on the applied development procedure. Although the development by enzymatic digestion is inferior to a development in organic solvents as revealed by sol–gel analysis and AFM, this process, in combination with cellulose based resists, paves the way towards a renewable and sustainable photolithographic procedure. In order to highlight the potential of this material for advanced patterning techniques, cellulose structures with sub-µm resolution were fabricated by means of TPA lithography. A potential application of this resist has been demonstrated by assembling an OTFT with an ultrathin patterned cellulose gate dielectric layer providing good performance (low interface trap density, low operation voltages, no hysteresis, appropriate field effect mobility). Considerably, the photopatterning capability of the gate dielectric promises fast, highly integrated, low-voltage organic electronic circuits, with a clearly simplified fabrication of via holes and therefore also a simplified design of circuits.

## Electronic supplementary material

Below is the link to the electronic supplementary material.

Supplementary material 1 (PDF 358 kb)
