# Supplementary material for: Photolithographic patterning of cellulose: a versatile dual-tone photoresist for advanced applications
Source: Cellulose (Lond). 2014 Oct 16;22(1):717–27. doi: 10.1007/s10570-014-0471-4 (PMC4579862; doi:10.1007/s10570-014-0471-4)
Supplement: Supplementary file 1 — Supplementary material 1 (PDF 358 kb) [file 10570_2014_471_MOESM1_ESM.pdf]

Supporting Information

Photolithographic Patterning of Cellulose - A Versatile Dual-tone Photoresist for Advanced Applications

Archim Wolfberger, Andreas Petritz, Alexander Fian, Jakob Herka, Volker Schmidt, Barbara Stadlober, Rupert Kargl, Stefan Spirk, Thomas Griesser

A. Wolfberger, J. Herka, T. Griesser

Chair of Chemistry of Polymeric Materials, University of Leoben, Otto Glöckel-Straße 2, 8700 Leoben, Austria

e-mail: thomas.griesser@unileoben.ac.at, phone: +43-3842-402-2358

A. Petritz, A. Fian, V. Schmidt, B. Stadlober

Materials-Institute for Surface Technologies and Photonics, Joanneum Research Forschungsgesellschaft mbH, Franz-Pichler-Straße 30, 8160 Weiz, Austria

R. Kargl

Faculty of Mechanical Engineering, University of Maribor, Smetanova 17, 2000 Maribor, Slovenia

S. Spirk

Institute for Chemistry and Technology of Materials, Graz University of Technology, Stremayrgasse 9, 8010 Graz, Austria

e-mail: stefan.spirk@tugraz.at, phone: +43-316-873-32284

Optical Spectroscopy

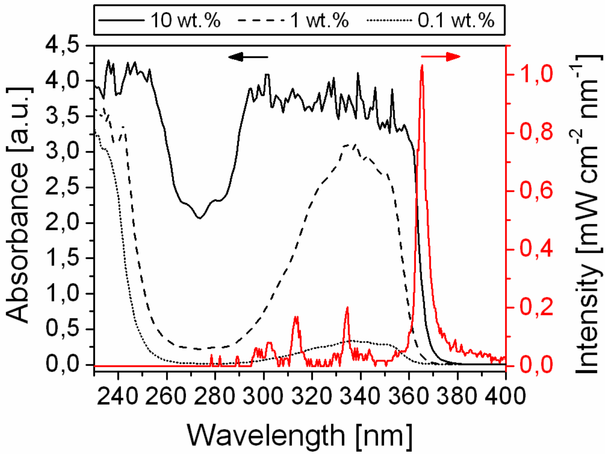

Fig. 1 Absorption spectrum of NHNA with a concentration of 0.1 wt.% (black solid line), 1 wt.% (black dashed line), and 10 wt.% (black dotted line) in chloroform and emission spectrum of the polychromatic UV-source, used for the irradiation experiments (red solid line)

Supplementary AFM Measurements

The rms roughness R_q of the differently treated samples was additionally determined by means of atomic force microscopy. Fig. 2 shows the corresponding AFM micrographs and line roughness profiles for TMSC films, containing 2 wt.% NHNA, after each treatment step. The rms roughness values of non-illuminated areas and illuminated areas do not differ significantly and exhibit values of 0.70 ± 0.04 and 0.78 ± 0.05 nm, respectively. After development in chloroform, the rms roughness of the remaining cellulose surface was determined as 0.75 ± 0.05 nm, which is still within the range of variation for individually prepared TMSC films, illustrating that the development step in chloroform does not significantly influence the roughness of the remaining cellulose features. The remaining areas of photopatterned TMSC films which were enzymatically digested and regenerated to cellulose show an increase of the rms roughness to 1.82 ± 0.02 nm, indicating that a positive type development with enzymes and hydrochloric acid negatively influences the surface properties to some degree under the chosen conditions.

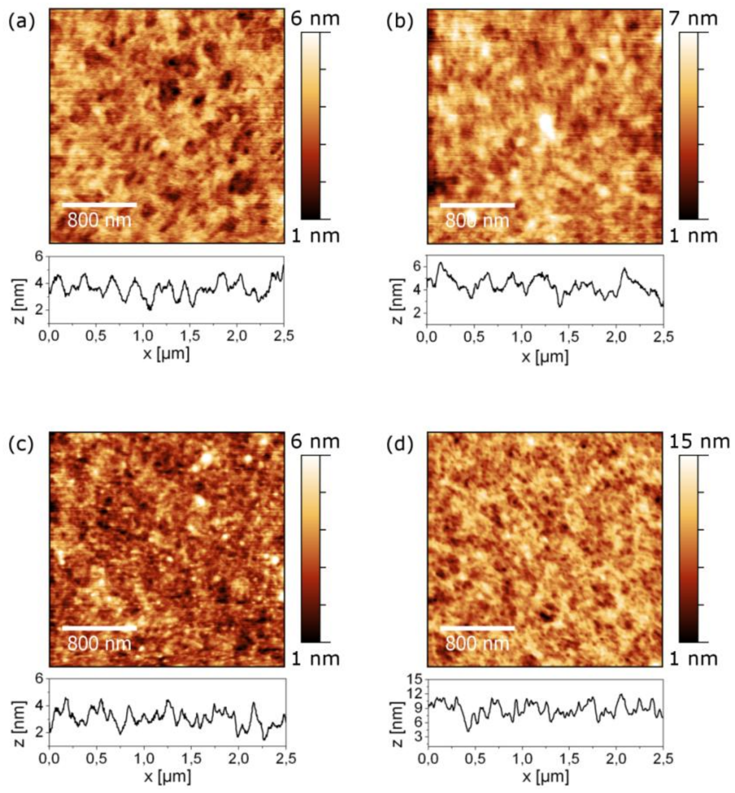

Fig. 2 AFM micrographs and corresponding line roughness of TMSC films, containing 2 wt.% NHNA before (a) and after (b) illumination with polychromatic UV light (E = 5.2 J cm^{-2}), followed negative type development (c) and positive type development (d)

XPS Measurements

In order to examine the chemical composition of TMSC surfaces prior to and after UV-illumination, X-ray photoelectron spectroscopy (XPS) was conducted. A comparison of C_{1s} detail spectra of pristine TMSC films with TMSC films, containing 2 wt.% NHNA which were exposed to UV light with an irradiation dose of 5.2 J cm^{-2} shows a significant reduction of the Si-C signal at 284.6 eV in relation to the C-O signal at 286.9 eV. Also a decrease of the total Si content from 11.7 at.% to 2.7 at.% (Fig. 3) can be observed, which is in good agreement with the conducted FTIR measurements.

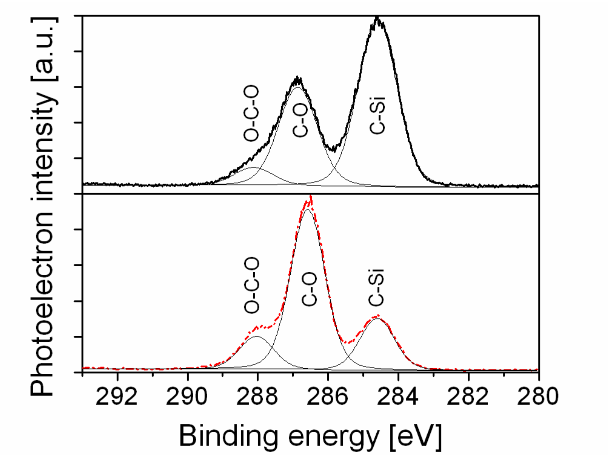

Fig. 3 XPS C_{1s} detail spectra of TMSC on Au (black solid line) and TMSC, containing 2 wt.% NHNA on Au after UV illumination (red dotted line)

Determination of the Surface Energy

The surface energy of the photopatternable polymer was determined via the Owens-Wendt-Rabel-Kaelble method, using contact angles of different liquids with known disperse and polar fractions of the surface tension.[1] Fig. 4 shows the calculated surface energies of TMSC films, containing 2 wt.% NHNA after a UV-illumination with different irradiation doses. The photochemical regeneration of TMSC to cellulose strongly affects the wettability of the layer, induced by the cleavage of the trimethylsilyl moieties, resulting in the generation of polar hydroxyl groups. The wettability of the TMSC surfaces strongly increases with higher irradiation doses. A reduction of the contact angle of H_2O from 99° ± 0.5° to 27° ± 0.5° after UV-irradiation with a dose of 16 J cm^{-2} is observed, which indicates that the photoinduced desilylation reaction leads to a more polar, hydrophilic surface. Non-illuminated TMSC films can be regarded as a low energy surface material with $\gamma = 22.7 \pm 0.4$ mN m^{-1} separable in a

polar component $\gamma_P = 1.2 \pm 0.1$ mN m^{-1} and a dispersive component $\gamma_D = 21.4 \pm 0.3$ mN m^{-1}, whereas UV-illumination increases the surface energy. After UV-irradiation with a dose of 16 J cm^{-2} a high surface energy of $\gamma = 73.3 \pm 0.3$ mN m^{-1}, $\gamma_P = 26.5 \pm 0.2$ mN m^{-1}, $\gamma_D = 46.7 \pm 0.1$ mN m^{-1} is observed.

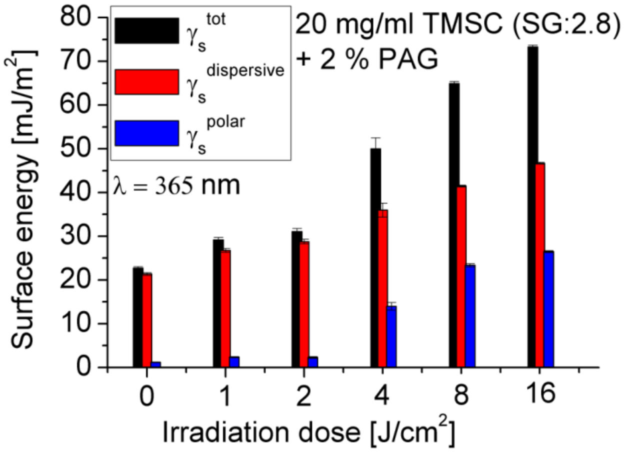

Fig. 4 Calculated surface energy of TMSC films, containing 2 wt.% NHNA after UV-illumination with different irradiation doses

Photopatterning of Ethyl Acetate Soluble TMSC ($DS_{Si} = 1.5$)

In order to further demonstrate the possibility of using more eco-friendly solvents for the photopatterning process, our approach has been applied to TMSC with a DS_{Si} of 1.5, which is soluble in ethyl acetate. The superiority of ethyl acetate over halogenated, aromatic or even non-halogenated hydrocarbons in terms of environmental-, health- and safety considerations has already been reported previously.[2] In these experiments, TMSC films were fabricated by spin coating (v = 2000 rpm, a = 1000 rpm s^{-1}) from ethyl acetate solutions (20 mg ml^{-1} TMSC, containibg 5 wt.% NHNA) onto silicon wafers. Photolithographic patterning was carried out with a mask aligner (500 W HgXe, SUSS, MJB4) equipped with a filter transmissive for wavelengths in the range of 365 nm (E = 5.2 J cm^{-2}). Subsequently, the development was performed in ethyl acetate for 15 min. Fig. 5 shows optical micrographs of the TMSC film after patterned illumination (left) and after development (right). It has to be mentioned that the patterning parameters (illumination dose, development time etc.) were not optimized for that particular patterning experiment. However, these results reveal the applicability of this approach for TMSC with various DS_{Si}.

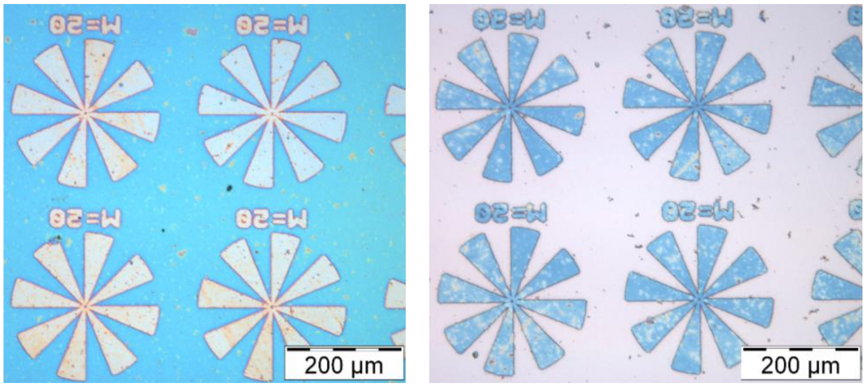

Fig. 5 Optical micrographs of TMSC (DS_{Si} = 1.5) films after patterned illumination (left, E = 5.2 J cm^{-2}) and after subsequent development in ethyl acetate (right)

[1] D. Owens and R. Wendt, *J. Appl. Polym. Sci,.* 1969, **13**, 1741.

[2] C. Capello, U. Fischer and K. Hungerbühler, *Green Chem.,* 2007, **9**, 927.
